# A home-based exercise program for children with congenital heart disease following interventional cardiac catheterization: study protocol for a randomized controlled trial

**DOI:** 10.1186/s13063-016-1773-7

**Published:** 2017-01-23

**Authors:** Qing Du, Yasser Salem, Hao (Howe) Liu, Xuan Zhou, Sun Chen, Nan Chen, Xiaoyan Yang, Juping Liang, Kun Sun

**Affiliations:** 1 Department of Rehabilitation Medicine, Xin Hua Hospital Affiliated to Shanghai Jiao Tong University School of Medicine, Shanghai, 200092 China; 2 Department of Physical Therapy, University of North Texas Health Science Center, Fort Worth, TX USA; 3 Department of Pediatric Cardiology, Xin Hua Hospital Affiliated to Shanghai Jiao Tong University School of Medicine, Shanghai, 200092 China

**Keywords:** Congenital heart disease, Cardiac catheterization, Children, Motor development, Home-based exercise

## Abstract

**Background:**

Cardiac catheterization has opened an innovative treatment field for cardiac disease; this treatment is becoming the most popular approach for pediatric congenital heart disease (CHD) and has led to a significant growth in the number of children with cardiac catheterization. Unfortunately, based on evidence, it has been demonstrated that the majority of children with CHD are at an increased risk of “non-cardiac” problems. Effective exercise therapy could improve their functional status significantly. As studies identifying the efficacy of exercise therapy are rare in this field, the aims of this study are to (1) identify the efficacy of a home-based exercise program to improve the motor function of children with CHD with cardiac catheterization, (2) reduce parental anxiety and parenting burden, and (3) improve the quality of life for parents whose children are diagnosed with CHD with cardiac catheterization through the program.

**Methods/design:**

A total of 300 children who will perform a cardiac catheterization will be randomly assigned to two groups: a home-based intervention group and a control group. The home-based intervention group will carry out a home-based exercise program, and the control group will receive only home-based exercise education. Assessments will be undertaken before catheterization and at 1, 3, and 6 months after catheterization. Motor ability quotients will be assessed as the primary outcomes. The modified Ross score, cardiac function, speed of sound at the tibia, functional independence of the children, anxiety, quality of life, and caregiver burden of their parents or the main caregivers will be the secondary outcome measurements.

**Discussion:**

The proposed prospective randomized controlled trial will evaluate the efficiency of a home-based exercise program for children with CHD with cardiac catheterization. We anticipate that the home-based exercise program may represent a valuable and efficient intervention for children with CHD and their families.

**Trial registration:**

http://www.chictr.org.cn/ on: ChiCTR-IOR-16007762. Registered on 13 January 2016.

**Electronic supplementary material:**

The online version of this article (doi:10.1186/s13063-016-1773-7) contains supplementary material, which is available to authorized users.

## Background

Congenital heart disease (CHD) is one of the most common structural abnormalities, occurring in 9 of every 1000 live births [1, 2]. Over the last three decades, dramatic changes have occurred in pediatric cardiac catheterization, and cardiac catheterization is now the main procedure for pediatric cardiac disease. In comparison with traditional cardiac surgical procedures (open heart surgery), therapeutic cardiac catheterization has several advantages, including being simpler and safer, and it is associated with an improved outcome [3]. Hence, pediatric therapeutic cardiac catheterization has increased recently because of numerous innovative catheter techniques, the increased number of persons and centers using these techniques, and the increased number of lesion types thought to be amenable to catheter therapy. It has been considered as the regular procedure clinically in dealing with patent ductus arteriosus, pulmonary stenosis, ventricular septal defect, and atrial septal defect [4].

Progress in medical diagnosis and new surgical techniques imply that the majority of children with complex CHD (80%) now enter adulthood successfully [5–7]. With increased survival rates, emerging evidence has highlighted that “non-cardiac” problems have increased rapidly in survivors [6, 8]. Studies have demonstrated that abnormal hemodynamics during fetal gestation and hypoxia in utero might play an important role in the risk of long-term adverse neurological outcomes in children with critical CHD [9, 10]. A distinctive pattern of neurodevelopmental and behavioral impairments has been noticed over the years; these are characterized by delayed motor development, cognitive impairments, and other abnormal growth [11, 12]. Many school-aged survivors adapt poorly to their school life because of low physical activity levels and poor academic performance, and these problems may persist into young adulthood, leading to a low quality of life for these children and their families [13].

Motor delays are common in children with CHD because of various reasons. Studies demonstrate that cardiac problems are not the only reason responsible for development delays [11, 14–16]; overprotection of parents or main caregivers is also a factor. Parents are the most concerned caregivers for their children, and overprotection of children with CHD has been observed with most parents and teachers. These attitudes and the anxiety of overprotective parents might restrict the physical activity of their children and reduce their children’s exposure to their peers, and this, in turn, might influence the social competence and motor development of these children. As a result, children may develop a sedentary lifestyle, and this may lead to increased risks of additional cardiovascular diseases and complications [17]. Without intervention, these development deficits may persist into adolescence and adulthood [18]. Mothers of children with CHD are more concerned and anxious about their children’s behaviors than parents of children without such health issues [19]. They often exaggerate the risk of adverse events or medical prognoses of CHD, and they may underestimate the adaptability of their children. The consequence of such overprotection may be reduced physical, emotional, psychosocial, or cognitive functioning in their children [11, 20].

A few studies have developed interventions to improve growth development in children with CHD, but there is still limited evidence in the literature to support the potential benefits of rehabilitation for these children [21–25]. Only one study implemented a home-based training program for 20 toddlers, aged 12 to 26 months, after either a superior cardiopulmonary connection procedure or an arterial switch operation. The study revealed that a home-based training program could improve motor abilities and increase children’s rates of development to age-appropriate norms [25]. But these studies focus on complex CHD. Even though cardiac catheterization is a minimally invasive procedure, it causes damage to the body; moreover, the patients are very young, with poor psychology and compliance, which make post-surgery management difficult. It is reported that a better inpatient environment with enough emotional support for the parents during the pre- and post-procedure phase, as well as exercise therapy for the children post-procedure, could relieve pain and reduce complications [26]. Overall, little research relates to growth in the development of therapeutic intervention for CHD with cardiac catheterization.

This parallel randomized controlled trial aims to evaluate the efficacy of a home-based exercise program and whether it could improve the motor abilities of children with CHD with cardiac catheterization, reduce parental anxiety and parenting burden, and improve the quality of life for the children’s parents through the program, which may represent a valuable and efficient intervention for children with CHD and their families.

## Methods/design

### Aims

The aims of this research study are as follows:

1. To evaluate whether a home-based exercise program may improve motor abilities in CHD children with cardiac catheterization
2. To identify whether a home-based exercise program may reduce parental anxiety and caregiver burden and promote parents’ quality of life.

### Study design

Children with CHD who are planning to perform a cardiac catheterization procedure will be recruited from the Department of Pediatric Cardiology, Xinhua Hospital affiliated to Shanghai Jiao Tong University School of Medicine, China.

**Fig. 1:**
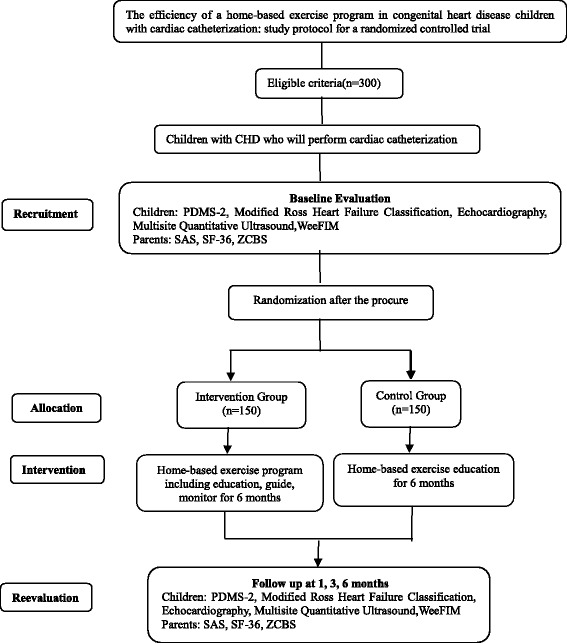
Flow diagram showing home-based exercise program for children with CHD following interventional cardiac catheterization

The study will be implemented at the Shanghai Jiao Tong University School of Medicine. Before participation in the study, parents/legal guardians will be asked to sign a written informed consent. First, patients who meet the inclusion criteria will be recruited for the study through echocardiography results. Second, patients will undergo baseline evaluations including motor abilities, cardiac function, modified Ross score, sound of speed at the tibia, and functional independence level, and their parents will complete three questionnaires about their anxiety levels, quality of life, and caregivers’ burden. Children with CHD will be allocated by a physician using computer-generated block randomization into two groups: an intervention group and a control group after the catheterization process. The allocations will be concealed in sequentially numbered, opaque, sealed envelopes through a signature across the sealing point. A trained research assistant, who will be blinded to the allocation, will enroll patients and assign them to interventions. All evaluations will be carried out by the evaluation team. Another team, composed of experienced pediatric physiotherapists, will teach parents the home-based exercises, remind them to perform the exercises, and provide valuable information about the details of the exercises. The evaluation team will be blinded in this program; however, the intervention team will not be blinded, as they must communicate with parents and offer informed consent in order to obtain the parents’ signatures. The intervention group will receive a home-based exercise program, while the control group will only receive home-based education (see Fig. 1). All children and parents will be evaluated by the trained evaluation team before the procedure and 1, 3, and 6 months after the cardiac catheterization. Micro Message Public Platform dissemination and collaboration with staff of the Department of Pediatric Cardiology was established to facilitate enrollment to reach the target sample size. All parents will be added into a CHD group of the Micro Message Platform created by the intervention team to remind them to bring their children for clinic visits in order to promote patient retention.

The intervention group will implement a home-based exercise program, while the control group will receive home-based exercise education. Both groups will continue with their routine activities, but they will not be able to attend any other formal exercise program.

**Fig. 2:**
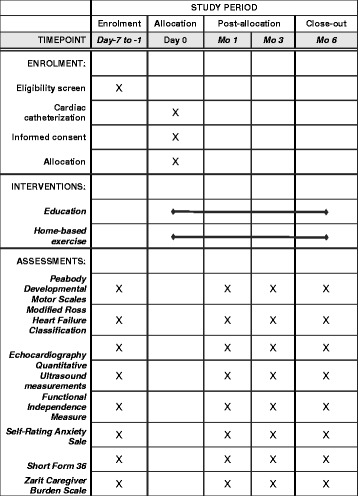
Time schedule of enrollment, assessments, and interventions

This study design follows the SPIRIT guidelines (see Fig. 2 and Additional files 1 and 2).

### Participants

#### Inclusion criteria

Children will be selected to participate in this study according to the following inclusion criteria: (1) echocardiography diagnosis of simple CHD with patent ductus arteriosus, pulmonary stenosis, ventricular septal defect, or atrial septal defect; (2) age 0 to 5 years; and (3) planned to undergo cardiac catheterization.

#### Exclusion criteria

Children will be excluded from participation in this study according to the following exclusion criteria: (1) arrhythmia; (2) CHD combined with other genetic disorders; (3) other congenital deformities; (4) liver or kidney diseases; (5) heart failure with a modified Ross score of 3 points or more; (6) history of heart surgery except cardiac catheterization; (7) operation on other organs; (8) previous rehabilitation treatment; (9) illnesses that may preclude the child from participation in the study as identified by the study physician.

### Withdrawal criteria and management

Children with CHD and their families will be allowed or be asked to withdraw from the study in the event of the following:

1. The child and his/her family make such a request.
2. The child has an adverse effect/event related to participation in the study.

### Intervention

The home-based exercise program will consist of home-based exercise education, home-based exercise, and home-based exercise supervision.

1. Home-based exercise education: The intervention team will explain the results of the developmental tests of their children to each family and emphasize the importance of home-based exercise for their children. Then, parents will receive a home-based physical activity brochure, and follow a Micro Message Public Platform that will share various forms of CHD knowledge twice monthly including exercise, education materials, and general care of children with CHD. It will also provide the exercise guide and tools for outpatient appointments. Parents will be asked to take their children to perform daily outdoor activities.
2. Home-based exercise: The home-based exercise program will be adopted from the motor activities program of Peabody Motor Development [27]. The home-based exercise program will be designed by the pediatric cardiologist, rehabilitation physician, and intervention team with input from the parents. The home-based exercise program will be individualized to each child’s developmental age, severity, and degree of developmental delay. The baseline assessment results will be used to identify age-appropriate skills that the children have not yet mastered. The exercise program will be designed so that parents can choose how to incorporate these activities into their daily schedules and preferred behaviors. First, a member of the intervention team will provide the parents with one-on-one rehabilitation program training until the parents master the skills. In addition, the parents will be given a home-based game reader and the Micro Message Public Platform to guide them. At least one of the children’s parents will be asked to complete the entire exercise program, and the rest of the family members must agree and support the home-based exercise program. The intervention team will maintain contact with the parents by phone to provide the exercise guide.

#### Outline of the home-based exercise program

The outline of the home-based exercise program will differ by developmental age:

1. Age 0–6 months
  - Developmental activities: activities/games with different postures, such as head lifting, support in prone position, hand, or elbow support, etc. For example, the infant could lie on the mother’s leg in a prone position; the mother could shake a soundtoy over the infant’s head to induce the infant to lift his/her head or hand; the infant can also lie on a big ball in a prone position
  - Passive exercise: stretching the infant’s limbs and shoulder, and wrist and leg manipulation by the parents, such as clapping or nudging the infant’s feet.
*Parents will implement the rehabilitation program at home over a 6-month period; the total daily time request will be 30 minutes for no less than 5 days per week.*
2. Age 7–12 months
  - Developmental activities: activities in different positions (prone, sitting, crawling, creeping, kneeling, and standing)
  - Passive exercise: stretching the infant’s limbs and shoulder, and wrist and leg manipulation by the parents, like the baby’s feet touching the mother’s feet with bending and extending movements, and stepping on a bicycle.
*Parents will implement the rehabilitation program at home over a 6-month period; the total daily time request will be 30 minutes for no less than 5 days per week.*
3. 13–24 months
  - Postural training: kneeling and standing
  - Flexibility training: active stretching of the upper and lower limbs, chest expansion, and shoulder, wrist, and leg movement
  - Breathing exercises: abdominal respiration, resisted breathing, deep breathing, and blowing bubbles and pinwheels
  - Developmental activities: walking, stair activities, stepping activities, and throwing a ball
  - Aerobic endurance training: swimming, riding a bike, and walking.
*Parents will implement the rehabilitation program at home over a 6-month period; the total daily time request will be 30 minutes for no less than 5 days per week.*
4. 25–60 months
  - Postural training: single-leg standing, standing on tiptoe, single-leg jumping, such as jumping following a rope with snake shapes, rope skipping, or standing on a soft cushion
  - Flexibility training: active stretching of the upper and lower limbs, chest expansions, and shoulder, wrist, and leg movements
  - Breathing training: abdominal respiration, resistant breathing, deep breathing, and blowing bubbles and pinwheels
  - Muscle strength training: pulling elastic bands with the upper limbs, squatting down and standing up, straight-leg raising movements, and gluteus training, like hiding in a big box, and inducing the child out with preferred toys
  - Developmental activities: climbing upstairs and coming downstairs, stepping activities, and throwing and kicking a ball
  - Aerobic endurance training: swimming, riding a bike, walking, jogging, and running to catch things with a crossing obstacle.
*Parents will implement the rehabilitation program at home over a 6-month period; the total daily time request will be 30 minutes for no less than 5 days per week.*

#### Safety supervision of home-based exercise training

Researchers will provide a portable device to parents that can be used to detect the blood oxygen saturation and heart rates of children with CHD. The heart rates of children with CHD will be maintained in a targeted range (60–80% of maximum heart rate) throughout the training. The training will stop if the child exceeds the maximum heart rate. The maximum heart rate will be calculated by a physiatrist according to the child’s age.

#### Compliance supervision

The intervention team will remind parents to carry out the exercise program and monitor each child’s progress through a Micro Message Public Platform or phone call one or two times weekly, and will also help them schedule rehabilitation evaluation appointments.

### Control group

The intervention team will explain the children’s evaluation results and share home-based exercise education with parents at baseline assessments. Home-based physical activity education will be given to the parents, but they will not receive the rehabilitation guide.

### Outcome measures

All assessments will be undertaken before the procedure and at 1, 3, and 6 months after the procedure.

### Primary outcome measures

#### Motor ability quotient

The Peabody Developmental Motor Scales, 2nd edition (PDMS-2) [28] will be used to assess each child’s motor development. The PDMS-2 is a performance-based tool used to assess motor development in both clinical and research settings. The PDMS-2 measures development in two domains, gross motor and fine motor, and it incorporates both quantitative and qualitative rating criteria. It consists of six sub-tests: reflexes, stationary, locomotion, object manipulation, grasping, and visual-motor integration. Sub-test scores are standardized by age and combined to calculate gross, fine, and total motor quotients. The raw scores for each of the sub-tests will be converted to age-equivalent scores, percentile ranks, standard scores, and composite scores. The PDMS-2 is a valid and reliable tool to assess motor development in children, and has excellent intra-rater reliability.

### Secondary outcome measurements

#### Ross score

The Modified Ross Heart Failure Classification will be used to assess children’s cardiac functions [29]. It is used to assess the cardiac functioning of children aged 0–14 years, including their history (diaphoresis, tachypnea) and physical examination (respiratory rate, heart rate, hepatomegaly size); the total scores range from 0 to 12 as follows: 0–2 (no congestive heart failure), 3–6 (mild congestive heart failure), 7–9 (moderate congestive heart failure), and 10–12 (severe congestive heart failure).

#### Index of echocardiography

The left ventricular diastolic diameter, left ventricular systolic diameter, and left ventricular wall thickness will be measured before and after the procedure to monitor the inner diameter changes in cardiac chamber. Function change of left and right ventricles, pulmonary artery systolic pressure, and pulmonary valve pressure difference will also be tested. The left ventricular end-systolic volume, left ventricular end-diastolic volume, and left ventricular short axis shortening rate are used to evaluate the left ventricular function. The tricuspid valve systolic peak velocity is used to check the right ventricular function. Measurements of highest velocity and defect size will be recorded before the procedure, and correct location and residual shunt will be verified by a cardiologist using echocardiography [4].

#### Speed of sound at the tibia

Speed of sound (SOS) will be evaluated using Quantitative Ultrasound measurements (Sunlight Omnisense TM7000, Petah Tikva, Israel) by the same trained rehabilitation physician. Each subject is seated close to the examination table and the patient’s non-dominant leg is rested. After introducing the water-soluble coupling gel, the probe moves across the tibia plane, searching for the site with a maximal reading. The measurement site is defined as the distal one-third of the tibia. The SOS is influenced by the bone minerals (major factor), bone thickness, microstructure, and skeletal elasticity [30].

#### Functional independence level

The WeeFIM (functional independence measure) instrument is a useful pediatric functional independence assessment tool for children aged 6 months to 7 years and for children with developmental disabilities aged 6 months to 21 years. It is an 18-item, 3-domain questionnaire that measures a child’s consistent performance in essential daily functional skills. Three main domains (self-care, mobility, and cognition) are assessed by interviewing or by observing a child’s performance of a task to criterion standards. Each item is rated on a 7-point ordinal scale ranging from 7 (complete independence) to 1 (total assistance). The WeeFIM is a psychometrically sound instrument in terms of its reliability, validity, and responsiveness [31]. Studies have already demonstrated that the WeeFIM can be used as a functional independence measure for Chinese children [32].

#### Anxiety

The Self-Rating Anxiety Sale (SAS) will be used to assess parents’ anxiety status. The SAS is a popular subject scale to measure anxiety. It has 20 items, scored as 1, 2, 3, or 4. Lower total scores mean lower anxiety: <50 (no anxiety); 51–60 (mild anxiety); 61–70 (moderate anxiety); >70 (severe anxiety) [33].

#### Quality of life

The Short Form 36 (SF-36) is used to evaluate parents’ quality of life; it is a widely used health status survey designed to assess quality of life by measuring the individual’s self-perception of his/her own health status with 8 multi-item scales, including physical functioning, physical role functioning, bodily pain, general health, vitality, social functioning, emotional role functioning, and mental health, and one single item of health transition. It can be used to assess the quality of life for patients with various diseases or people in general. The reliability, validity, and sensitivity of the Chinese (simple) SF-36v2 have been verified [34].

#### Caregiver burden

The Zarit Caregiver Burden Scale (ZCBS) is a widely used and valued assessment tool for caregiver burden, which was designed in line with Zarit’s Caregiver Burden measurement theory [35]. The ZCBS has two dimensions: personal strain and role strain, with a total of 22 items. Each item is rated on a 4-point scale, and higher scores represent a more serious burden: 0–20 (little or no burden); 21–40 (mild to moderate burden); 41–60 (moderate to severe burden); 61–88 (severe burden), corresponding to the subjective feeling.

### Sample measurement

GPower 3.1.9.2 will be used to perform the power calculations. The motor quotient of the PDMS-2 will be our primary outcome measurement.

The results of our pilot study showed that after 6 months of intervention, the motor quotient of the intervention group with patent ductus arteriosus on average was (94.33 ± 11.29), and that of the control group was (84.67 ± 6.11); therefore, the effect size was 1.06. Thus, as the α will be 0.05 and the β will be 0.05, each group should recruit 24 patients. Considering 10% potential attrition, 27 patients in each group with patent ductus arteriosus will be recruited.

The motor quotient of the intervention group with pulmonary stenosis on average after 6 months of intervention was (101.00 ± 9.90), and that of the control group was (89.00 ± 1.41); therefore, the effect size was 1.70. Thus, as the α will be 0.05 and the β will be 0.05, each group should recruit 11 patients. Considering 10% potential attrition, 13 patients in each group with pulmonary stenosis will be recruited.

The motor quotient of the intervention group with ventricular septal defect on average after 6 months of intervention was (95.00 ± 8.54), and that of the control group was (90.50 ± 7.78); therefore, the effect size was 0.55. Thus, as the α will be 0.05 and the β will be 0.05, each group should recruit 87 patients. Considering 10% potential attrition, 96 patients in each group with ventricular septal defect will be recruited.

The motor quotient of the intervention group with atrial septal defect on average after 6 months of intervention was (99.67 ± 5.43), and that of the control group was (90.80 ± 5.72); therefore, the effect size was 1.59. Thus, as the α will be 0.05 and the β will be 0.05, each group should recruit 12 patients. Considering 10% potential attrition, 14 patients in each group with atrial septal defect will be recruited.

### Data collection, management, and analysis

Data will be entered using EpiData software designed for this study. All data will be collected, typed, and analyzed by a statistician, who will be blinded during the trial. The main investigators will check the data every 2 weeks to ensure the quality. All statistical analyses will be performed using SPSS 20.0. Descriptive data will be presented as mean ± standard deviation. Considering that age may be a potential factor influencing the outcome measurement, a covariance analysis will be used to compare the effects between two groups. A *t* test will be used to compare changes in parent outcome measures in the two groups. Multiple linear mixed models will be used to analyze the relationships between the risk factors and the outcome measures. An intention-to-treat analysis will be used if participants are lost to follow-up. All statistical tests will be performed at a significance level of 0.05.

The parents will also be informed of this crucial aspect, and a member of the intervention team will be available any time the parents may need further information or clarification during the study period.

An interim analysis will be performed by the statistician on the primary endpoint; the statistician will be blinded for treatment allocation and will report to the main investigators. The main investigators will discuss the results of the interim analysis with the monitoring board. However, the trial will be terminated in case of harm. The criterion for stopping the trial for harm is as follows: a statistically significant difference in the primary outcome between the intervention group and a reasonable suspected causal relationship between the intervention and adverse events.

### Harms

If there is a reasonable suspected causal relationship with the intervention, the adverse events will be reported to the Ethics Committee to guarantee the safety of the participants. We consider that there will be no risks for either group (patients with or without intervention).

### Data monitoring and auditing

A monitoring board, including independent assessors (not involved in the study) from the Shanghai Jiao Tong University School of Medicine, will review all data and can conduct an audit of the trial at any time.

### Confidentiality

Only the main investigators will be allowed back-end EpiData software entry with passwords. All children with CHD will be identified by sex, birth date, and evaluation date, and will be assigned a trial number during and after the trial in accordance with personal data protection laws.

### Access to data

The main investigators will have the right to enter the final and complete trial dataset, and there is no contractual agreement to limit such access to all the investigators.

### Ancillary and post-trial care

After completing the trial, we will continue to evaluate and treat the patients in the future according to their parents’ wishes.

### Dissemination policy

The final results of the trial are planned to be published in a scientific journal and presented at medical conferences. The final reporting will follow the Consolidated Standards of Reporting Trials (CONSORT) Statement guidelines (http://www.consort-statement.org).

## Discussion

The home-based exercise program may not only contribute to an increase in the motor performance of children when the parents are included; it may also reduce unnecessary concerns [20, 25]. We will implement appropriate guidelines and supervision for the home-based exercise program; for example, we will call the parents to remind them to carry out the rehabilitation program weekly and to answer any questions or concerns. In addition, we will hand out brochures and disks to share exercise education materials. Our study may provide replicable evidence that a home-based exercise program can improve motor abilities in children with CHD and improve parental anxiety, caregiver burden, and the overall quality of life.

### Strengths and limitations

The first strength of our study is that it will be the first randomized controlled trial that focuses on home-based exercise for young children with CHD and cardiac catheterization. Second, except for the important elements of cardiac function, our study focuses more on growth development at an early stage for children with CHD. Third, we used GPower 3.1.9.2 software for the sample calculation to ensure its scientific validity. Fourth, our study will last for 6 months, with three re-evaluations, in order to update the patients’ recovery and development status in time. Fifth, our study develops a detailed and individual home-based exercise program for each patient, and provides a one-on-one guide for the parents, until they master the skills. Moreover, there is a close home-based monitoring process, which could ensure the quality of implementation of the home-based exercise program to a certain extent. Sixth, our intervention will not solely focus on the children, as their parents will also be educated in home-based exercise and other appropriate educational and care guidelines for children with CHD.

Several limitations exist in our trial: (1) the age of subjects is limited to 0 to 5 years; (2) we only recruit patients with cardiac catheterization for our trial; we will not group the children according to their specific CHD subtypes or the treatment approach to CHD; (3) we will not evaluate language and speech development or cognitive development directly, only motor development; thus, our trial cannot be used as a comprehensive evaluation of all types of home-based exercise programs for CHD children with cardiac catheterization; and (4) our study includes a short follow-up duration of 6 months.

In conclusion, our study design for delayed motor development of CHD children with cardiac catheterization developed a home-based exercise program as the main intervention approach after the procedure. It is crucial to address whether a home-based exercise program could improve the patients’ motor abilities and improve parental anxiety, burden, and quality of life. The findings will be beneficial for children with CHD and their families, research collaborators, physicians, and the general public.

### Trial status

Patient recruitment is ongoing. Recruitment of study participants commenced on 10 January 2016.

## Additional files

Additional file 1: SPIRIT 2013 checklist: Recommended items to address in a clinical trial protocol and related documents.* (DOC 132 kb)
Additional file 2: Table S1. World Health Organization Trial Registration Data Set. (DOC 45 kb)

## Abbreviations

CHD: Congenital heart disease
PDMS-2: Peabody Developmental Motor Scales-2
SOS: Speed of sound
WeeFIM: Functional independence measure instrument
