# Supplementary material for: A home-based exercise program for children with congenital heart disease following interventional cardiac catheterization: study protocol for a randomized controlled trial
Source: Trials. 2017 Jan 23;18:38. doi: 10.1186/s13063-016-1773-7 (PMC5259847; doi:10.1186/s13063-016-1773-7)
Supplement: Additional file 2: Table S1. — World Health Organization Trial Registration Data Set. (DOC 45 kb) [file 13063_2016_1773_MOESM2_ESM.doc]

**Additional file 2: Table S1** **World Health Organization Trial Registration Data Set (Version 1.2.1)**

| Item number | Item | Description | Addressed on page number |
| --- | --- | --- | --- |
| 1 | Primary registry and trial identifying number | Name of primary registry and the unique identifier assigned by the primary registry | 3 |
| 2 | Date of registration in  primary registry | Date when the trial was officially registered in the primary registry | 3 |
| 3 | Secondary identifying  numbers | Other identifiers, if any Universal Trial Number Identifiers assigned by the sponsor Other trial registration numbers issued by other registries Identifiers issued by funding bodies, collaborative research groups, regulatory authorities, ethics committees/institutional review boards, etcetera. | Not applicable |
| 4 | Sources of monetary or material support | Major sources of monetary or material support for the trial (for example, funding  agency, foundation, company, and institution) | 17 |
| 5 | Primary sponsor | Person, organization, group, or other legal entity that takes responsibility for initiating  and managing a study | 17 |
| 6 | Secondary sponsor(s) | additional persons, organizations, or other legal persons, if any, who have agreed with the primary sponsor to take on responsibilities of sponsorship | Not applicable |
| 7 | Contact for public queries | E-mail address, telephone number, and postal address of the contact who will respond to general queries, including information about current recruitment status | 2 |
| 8 | Contact for scientific queries | Name and title, e-mail address, telephone number, postal address, and affiliation of the principal investigator and e-mail address, telephone number, postal address, and affiliation of the contact for scientific queries about the trial (if applicable) | 2 |
| 9 | Public title | Title intended for the lay public in easily understood language 1 | 1 |
| 10 | Scientific title | Scientific title of the study as it appears in the protocol submitted for funding and ethical review; include trial acronym, if available | Not applicable |
| 11 | Countries of recruitment | Countries of recruitment Countries from which participants will be recruited | 6 |
| 12 | Health condition(s) or problem(s) studied | Primary health condition(s) or problem(s) studied (for example, depression, breast cancer, or medication error) | 5 |
| 13 | Intervention(s) | For each group of the trial, record a brief intervention name plus an intervention description name. For drugs, use the generic name; for other types of interventions, provide a brief descriptive name of the intervention. This name must be sufficiently detailed for it to be possible to distinguish between the groups of a study; for example, interventions involving drugs may include dosage form, dosage, frequency, and duration | 7-10 |
| 14 | Key inclusion and exclusion criteria | Inclusion and exclusion criteria for participant selection, including age and sex | 7 |
| 15 | Study type | Method of allocation (randomized/ nonrandomized) and blinding/masking (identify who is blinded) Assignment (for example, single group, parallel, crossover, or factorial) and purpose Phase (if applicable) For randomized trials - method of sequence generation  and allocation concealment | 5 |
| 16 | Date of first enrollment | Anticipated or actual date of enrollment of the first participant | 16 |
| 17 | Target sample size | Total number of participants to enroll | 13 and 16 |
| 18 | Recruitment status | Pending - participants are not yet being recruited or enrolled at any site Recruiting-participants are currently being recruited and enrolled Suspended - temporary halt in recruitment and enrollment  Complete - participants are no longer being recruited or enrolled Other |  |
| 19 | Primary outcome(s) | The primary outcome should be the outcome used in sample size calculations or the main outcome used to determine the effects of the intervention For each primary outcome, provide the following: Name of the outcome (do not use abbreviations) Metric or method of measurement used (be as specific as possible)  Time point of primary interest | 10 |
| 20 | Key secondary outcome(s) | As for primary outcomes, for each secondary outcome provide the following: Name of the outcome (do not use abbreviations) Metric or method of measurement used  (be as specific as possible) Time point of interest | 11 and 12 |
